# Predictors for good functional outcome after neurocritical care

**DOI:** 10.1186/cc9192

**Published:** 2010-07-20

**Authors:** Ines C Kiphuth, Peter D Schellinger, Martin Köhrmann, Jürgen Bardutzky, Hannes Lücking, Stephan Kloska, Stefan Schwab, Hagen B Huttner

**Affiliations:** 1 Department of Neurology, University of Erlangen, Schwabachanlage 6, 91054 Erlangen, Germany; 2 Department of Neuroradiology, University of Erlangen, Schwabachanlage 6, 91054 Erlangen, Germany

## Abstract

**Introduction:**

There are only limited data on the long-term outcome of patients receiving specialized neurocritical care. In this study we analyzed survival, long-term mortality and functional outcome after neurocritical care and determined predictors for good functional outcome.

**Methods:**

We retrospectively investigated 796 consecutive patients admitted to a non-surgical neurologic intensive care unit over a period of two years (2006 and 2007). Demographic and clinical parameters were analyzed. Depending on the diagnosis, we grouped patients according to their diseases (cerebral ischemia, intracranial hemorrhage (ICH), subarachnoid hemorrhage (SAH), meningitis/encephalitis, epilepsy, Guillain-Barré syndrome (GBS) and myasthenia gravis (MG), neurodegenerative diseases and encephalopathy, cerebral neoplasm and intoxication). Clinical parameters, mortality and functional outcome of all treated patients were analyzed. Functional outcome (using the modified Rankin Scale, mRS) one year after discharge was assessed by a mailed questionnaire or telephone interview. Outcome was dichotomized into good (mRS ≤ 2) and poor (mRS ≥ 3). Logistic regression analyses were calculated to determine independent predictors for good functional outcome.

**Results:**

Overall in-hospital mortality amounted to 22.5% of all patients, and a good long-term functional outcome was achieved in 28.4%. The parameters age, length of ventilation (LOV), admission diagnosis of ICH, GBS/MG, and inoperable cerebral neoplasm as well as Therapeutic Intervention Scoring System (TISS)-28 on Day 1 were independently associated with functional outcome after one year.

**Conclusions:**

This investigation revealed that age, LOV and TISS-28 on Day 1 were strongly predictive for the outcome. The diagnoses of hemorrhagic stroke and cerebral neoplasm leading to neurocritical care predispose for functional dependence or death, whereas patients with GBS and MG are more likely to recover after neurocritical care.

## Introduction

Within the last decades, specialized neurocritical intensive care units (NICU) have evolved from bigger, multi-disciplinary ICUs [1]. This specialization has led to a decrease in both in-hospital mortality and length of hospital stay without associated effects on readmission rates and long-term mortality [2]. Nevertheless, case fatality of patients admitted to NICUs is still high and the outcome often poor [3]. Yet, there are still little data on clinical parameters associated with long-term outcome after neurocritical care; aside from age, the major determinant for outcome, hospital length of stay and the diagnosis of stroke have been shown to negatively influence outcome [3].

In order to provide data that facilitate the assessment of long-term prognosis after neurocritical care we aimed to identify predisposing factors for a good functional recovery one year after treatment on a specialized neurocritical care unit.

## Materials and methods

### Patients and setting

The present analysis was based on patients who were admitted to our 10-bed NICU (University Hospital Erlangen, Tertiary University Hospital) in 2006 and 2007. Given a separate neurosurgical ICU on the same floor, patients with neurosurgical diseases such as traumatic brain injury are not treated in our NICU. Because there is an additional 14-bed stroke and intermediate care unit, according to an institutional protocol, all patients admitted to our NICU must fulfill at least one of the following criteria: requiring mechanical ventilation, intravenous catecholamines, extraventricular or lumbar drainages, or have a Glasgow Coma Scale (GCS) below or equal to nine points. Furthermore, patients with evidence of vasospasms were also treated in our NICU. According to an inter-institutional protocol, patients with subarachnoid hemorrhage (SAH) who were treated endovascularly were admitted to our NICU, whereas SAH patients who were treated surgically were admitted to the neurosurgical ICU. Likewise, patients with brain tumors at operable stages were treated neurosurgically; those who were not operable, were treated neurologically.

**Figure 1:**
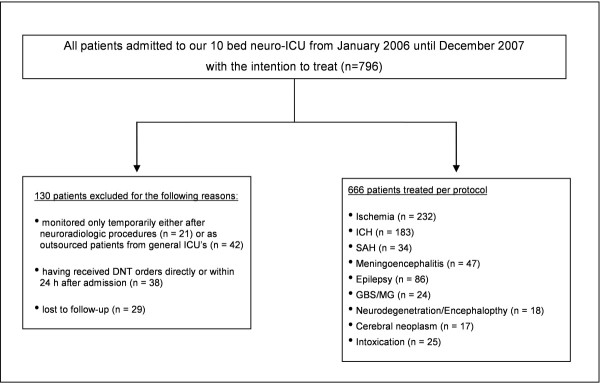
**Flowchart of patient selection**.

Seven-hundred and ninety-six neurological patients were admitted over the two-year period, representing our *intention-to-treat *cohort. Detailed data on this group are given in Figure 1 and Table 1. To more reliably analyze prediction of functional outcome we focussed on those patients who received specialized neurocritical care and excluded all patients who were set on *do not treat *(DNT) orders at admission. Patients who were set on DNT orders were most severely affected (for example, signs of herniation on admission because of massive ICH) or they had severe co-morbidities and did not consent to invasive critical care therapies. These patients did not receive any treatment, except for intravenous fluids and morphine, and were only admitted to our neurocritical care unit because of already having been intubated and ventilated prior to hospital arrival. We determined *a priori *to focus on treated patients only and thus excluded patients with early DNT orders from our analysis of functional outcome after specialized neurocritical care. Contrary to this, patients who received any other therapeutic procedure are not referred to as DNT and remained in our analysis. Furthermore, we excluded all patients who were monitored on our NICU only temporarily either as outsourced patients from other ICUs or because of elective neuroradiologic procedures; that is, patients who were monitored for only few hours until extubation after intracranial stenting or coiling (Figure 1). Moreover, patients that were lost to follow-up at one year after discharge were excluded (*n *= 29; the baseline clinical data as well as in-hospital mortality of patients lost to follow-up did not vary significantly from the cohort analyzed (data not shown). Overall, 666 patients remained for final analysis and we refer to this group as the *per protocol *cohort. The institutional review board approved the study and consent was obtained in written or oral form from all patients or their relatives/legal guardians.

**Table 1:** Demographic and clinical data

	All	Ischemia	ICH	SAH	Meningoencephalitis	Epilepsy	GBS/MG	Neurodeg/Encephalopathy	Neoplasm	Intoxication	Temporarily monitored
**n **(%)	733	247 (33.7)	210 (27.4)	38 (5.2)	49 (6.7)	93 (12.7)	25 (3.4)	22 (3.0)	22 (3.0)	27 (3.7)	**63**
**Age **(median, range)	67 (18 to 95)	72 (21 to 93)	70 (35 to 95)	56 (19 to 84)	63 (27 to 85)	59 (18 to 93)	58 (23 to 78)	66 (23 to 90)	65 (39 to 78)	53 (29 to 78)	**51 (33 to 68)**
**Female sex **(n, %)	350 (47.7)	111 (44.9 )	100 (47.6)	20 (52.6)	26 (53.1)	48 (51.6)	14 (56)	14 (63.6)	8 (36.4)	9 (33.3)	**26 (41.3)**
**Pre-hospital mRS 0 to 2 **n (%)	635 (86.6)	227 (91.9)	198 (94.3)	37 (97.4)	43 (87.8)	71 (76.3)	24 (96.0)	8 (36.4)	4 (18.2)	23 (85.2)	**47 (74.6)**
**Hospital length of stay **in days (median, range)	4 (0 to 87)	4 (0 to 57)	5 (0 to 53)	4 (0 to 63)	4 (0 to 84)	1 (0 to 87)	6 (0 to 57)	3 (1 to 50)	3 (0 to 17)	3 (0 to 19)	**1 (0 to 2)**
**Mechanichal ventilation **(n, %)	450 (61.4)	148 (59.9)	140 (66.7)	20 (52.6)	42 (85.7)	48 (51.6)	16 (64.0)	18 (81.8)	8 (36.4)	20 (74.1)	**18 (28.6)**
**Length of ventilation **(d) (median, range)	3 (0 to 83)	4 (0 to 43)	5 (0 to 53)	1(0 to 60)	4 (0 to 83)	0 (0 to 63)	6 (0 to 49)	3 (0 to 50)	0 (0 to 13)	3 (0 to 14)	**0 (0 to 1)**
**DNT **(n, %)	**38 (5.1)**	**9 (3.6)**	**22 (10.5)**	**3 (7.9)**	**0**	**0**	**0**	**2 (9.1)**	**2 (9.1)**	**0**	**0**
**Lost to follow-up **(n, %)	**29 (4.0)**	**6 (2.4)**	**5 (2.4)**	**1 (2.6)**	**2 (4.1)**	**7 (7.5)**	**1 (4)**	**2 (9.1)**	**3 (13.6)**	**2 (7.4)**	**16 (25.4)**
**In-hospital mortality **(n, %)	165 (22.5)	53 (21.5)	75 (35.7)	12 (31.6)	3 (6.1)	5 (5.4)	1 (4.0)	4 (18.2)	7 (31.8)	5 (18.5)	**0**
**Mortality after 1 year **(n, %)	292 (39.8)	100 (41.5)	121 (59.0)	18 (48.6)	6 (12.8)	14 (16.3)	1 (4.2)	11 (55.0)	15 (78.9)	6 (24.6)	**1 (2.1)**
**mRS 0 to 2 after 1 year **(n, %)	208 (28.4)	33 (13.7)	40 (19.5)	11 (29.7)	28 (59.6)	57 (66.3)	16 (66.7)	5 (25.0)	1 (5.3)	17 (68.0)	**38 (80.9)**

Demographic and clinical characteristics of all patients (*n *= 796) including a separate analysis for admission diagnosis (intention-to-treat cohort).

Patients who were excluded for the per protocol-analysis are highlighted in bold. (PS: All patients monitored only temporarily (right column) are not included in the overall numbers (left column). Patients lost to follow up are not included in mortality and functional outcome after one year.)

Abbreviations: ICH, intracranial hemorrhage; SAH, subarachnoid hemorrhage; GBS, Guillain-Barré syndrome; MG, myasthenia gravis; Neurodeg, neurodegenerative disease; n, number; d, days.

### Data collection and outcome analysis

The parameters of age, sex, pre-admission mRS, length of hospital stay (LOS; in days), diagnosis, duration time of ventilation (LOV; in days) and modified TISS-28 (Therapeutic Intervention Scoring System) [4] were obtained by reviewing the patients' hospital charts and institutional electronic databases. Mortality and functional outcome one year after discharge (as modified Rankin Scale (mRS)) were obtained by a mailed standardized questionnaire. In all cases in which this questionnaire did not return within six weeks, a standardized phone interview was conducted with the patients, or their closest relatives, respectively [5]. The telephone interviews were performed by one physician who was trained and certified for data collection on disability, quality of life, and the mRS.

Given the heterogeneous patient population we grouped the patients with respect to their diagnoses (ischemic stroke, intracranial hemorrhage (ICH), subarachnoid hemorrhage (SAH), epileptic seizures, meningoencephalitis, Guillain-Barré syndrome (GBS) and Myasthenia gravis (MG), neurodegeneration/encephalopathy, inoperable cerebral neoplasm, and intoxication). Functional outcome was defined as good (mRS 0 to 2; *independent*) or poor (mRS 3 to 6; *dependent or dead*). In addition, in-hospital mortality and the mortality rates one year after discharge were assessed.

### Statistical analysis

Statistical analyses were performed using the SPSS 17.0 software package (SPSS Inc., Chicago, IL, USA). Statistical tests were two-sided and the significance level was set at α = 0.05. The distribution of the data was assessed with the Kolmogorov-Smirnov test. Continuous and categorical variables are expressed as mean and SD, as median and range, or as percentage, as appropriate. Proportions between two groups were compared by using the χ2 test, Fisher's exact test or Mann-Whitney U test, as appropriate.

One stepwise forward inclusion multivariate logistic regression model was calculated for prediction of good functional outcome one year after neurocritical care including those parameters that showed at least a trend when being tested univariately (*P *< 0.1). Interaction terms did not reveal significant interaction between the variables. In the univariate and multivariate analyses, the parameters LOS and LOV were calculated as dichtomized variables (according to their median); however, LOV was also calculated as a continuous variable (that is, increasing days) in the univariate analysis. The modified TISS-28 score is given as value per day and was categorized (< 21, 21 to 40, > 40), as described previously [3].

## Results

### Analysis of all patients admitted to the neurocritical care unit (Intention-to-treat population)

The demographic and clinical characteristics of all 796 patients are given in Table 1. The analysis according to admission diagnoses revealed that nearly 60% of all patients suffered from stroke (ischemic stroke: *n *= 247; 31% and ICH: *n *= 210; 26%). Patients were diagnosed with SAH in 5% (*n *= 38), epileptic seizures in 12% (*n *= 93), meningoencephalitis in 6% (*n *= 49), Guillain-Barré-Syndrome and myasthenia gravis in 3% (*n *= 25), neurodegenerative diseases and encephalopathy in 3% (*n *= 22), cerebral neoplasm in 3% (*n *= 22), and intoxications in 3% (*n *= 27). The remaining 63 patients were patients outsourced from general ICUs due to space limitations as well as patients temporarily monitored after neuroradiological procedures. The median length of stay was four days (0 to 87 days). The median length of ventilation was three days (0 to 83 days). The median modified TISS-28 score on Day 1 was 38 (18 to 71), the median TISS-28 score at discharge was 19 (15 to 43). When only focussing on treated patients, the in-hospital mortality amounted to 22.5%, and mortality rate after one year was 39.8%. Including patients who were set on DNT orders, the in-hospital mortality rate amounted to 27.7%, and the one-year mortality rate was 45.0%. For more detailed data please refer to Table 1.

### Analysis of patients receiving specialized neurocritical care (per protocol population)

**Figure 2:**
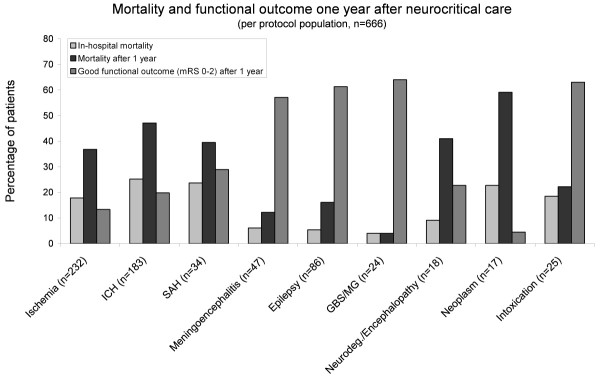
**Functional status after one year, in-hospital mortality and mortality after one year for all patients treated per protocol (*n *= 666)**.

Figure 2 shows data on in-hospital mortality, mortality after one year, and functional outcome one year after neurocritical care. Overall in-hospital mortality was 19.1%, and overall mortality after one year was 38.1%. Proportional to the patient numbers, ischemic stroke, ICH, SAH, cerebral neoplasm, and neurodegenerative diseases revealed the highest frequency of in-hospital and long-term mortalities compared to the remaining patients (*P *< 0.01). Overall, 31.2% of the per-protocol population achieved a favorable functional long-term outcome. Good functional outcome was achieved significantly more often in patients with meningoencephalitis, epilepsy, GBS/MG, and intoxication (*P *< 0.01).

### Prediction of good functional long-term outcome after neurocritical care

The logistic regression analysis of all patients who received specialized neurocritical care for prediction of a good functional outcome one year after discharge is shown in Table 2. After adjustment, the diseases GBS and MG were independently related to a good long-term outcome, whereas age, LOV, and TISS-28 score on Day 1 as well as the diagnoses ICH and cerebral neoplasm were predisposing factors for an unfavorable outcome.

**Table 2:** Predictors for functional outcome

	Good outcome (mRS ≤ 2)		
		
	Exp(Coef)	95% CI	*P*-value
**Univariate**			

Age	0.834	0.794 to 0.872	< 0.0001
SEX (female)	1.265	0.554 to 1.864	0.57645
**Hospital LOS**	**0.759**	**0.281 to 0.866**	**0.04397**
**Length of ventilation**	**0.410**	**0.113 to 0.641**	**0.01202**
*Length of ventilation (per increasing day)*	*0.974*	*0.913 to 1.061*	*0.09441*
TISS-28 on Day 1			
**< 21**	**2.746**	**1.935 to 4.391**	**0.00666**
**20 to 40**	**1.273**	**0.764 to 1.812**	**0.21932**
**> 40**	**0.715**	**0.621 to 0.944**	**0.00181**
TISS-28 at discharge			
**< 21**	**2.187**	**1.453 to 3.812**	**0.01215**
**20 to 40**	**1.234**	**0.218 to 2.187**	**0.23156**
**> 40**	**0.711**	**0.451 to 0.857**	**0.00017**
**Ischemia**	**0.724**	**0.485 to 0.932**	**0.04275**
**ICH**	**0.743**	**0.573 to 0.935**	**0.02046**
SAH	0.636	0.273 to 1.198	0.63532
Meningoencephalitis	1.412	0.996 to 3.238	0.12432
Epilepsy	1.433	0.233 to 2.346	0.73255
**GBS/MG**	**3.623**	**1.124 to 13.327**	**0.00145**
*Neurodeg./Encephalopathy*	*0.680*	*0.274 to 1.019*	*0.05723*
**Neoplasm**	**0.692**	**0.371 to 0.856**	**0.00039**
**Intoxication**	**5.809**	**1.832 to 7.483**	**0.03881**

**Multivariate**			

**Age**	0.786	0.435 to 0.823	0.00245
Hospital LOS	0.509	0.272 to 1.279	0.17647
**Length of ventilation**	**0.681**	**0.475 to 0.912**	**0.00354**
**TISS-28 on Day 1 > 40**	**0.815**	**0.578 to 0.931**	**0.00187**
Ischemia	0.345	0.245 to 1.101	0.11458
**ICH**	**0.643**	**0.218 to 0.877**	**0.03874**
**GBS/MG**	**2.215**	**2.006 TO 3.214**	**0.03329**
Neurodeg./Encephalopathy	0.705	0.297 to 1.354	0.15478
**Neoplasm**	**0.687**	**0.354 to 0.934**	**0.04875**
Intoxication	1.399	0.964 to 2.648	0.27261

Univariate and multivariate regression analysis for parameters predicting a good functional outcome (mRS 0 to 2) one year after neurocritical care. Analysis of all patients receiving specialized neurocritical care (*n *= 666). Parameters that reached significance (*P *< 0.05) are expressed in bold. Parameters that showed a statistical trend (*P *< 0.1) in the univariate analysis are expressed in italics.

Abbreviations: CI, confidence interval; LOS, length of stay; ICH, intracranial hemorrhage; SAH, subarachnoid hemorrhage; GBS, Guillain-Barré syndrome; MG, myasthenia gravis; Neurodeg, neurodegenerative disease

## Discussion

First of all it has to be noted that neurocritical care medicine deals with severely ill patients with a highly constricted capability of regeneration of neurons of the central nervous system. Hence, neurological syndromes due to neurovascular and inflammatory causes, which represent the majority in this study and result in the necessity of intensive care only show a limited capacity of complete recovery, whereas diseases without affection of neurons, that is, due to autoimmune causes, recovery may not be limited [6]. Nonetheless, specialized neurocritical care is justified as a number of specific treatment regimens have emerged over the past years. Examples for specific neurocritical care therapies include hemicraniectomy for treatment of space-occupying large cerebral infarctions [7] as well as continuous intracranial pressure and oxygen monitoring with intraparenchymal probes [8]. Patients with basilar artery and carotid artery-T occlusion may receive interventional treatment with a combined approach of intravenous and intraarterial thrombolytics [9] with or without use of recanalisation devices [10]. ICH patients with intraventricular hemorrhage may undergo special treatment with extraventricular drainages, intraventricular thrombolysis and lumbar drainage for communicating hydrocephalus [11-13]. Both ischemic and hemorrhagic stroke can be treated with endovascular cooling to reduce edema formation and to reduce further impairment of so far healthy brain tissue [14]. Furthermore, patients with GBS are treated with plasma exchange, intravenous immunoglobulins, ventilation and external pacemakers for severe autonomic dysfunction [15,16]. However, the benefit of some of these neurointensive procedures has not been shown in large randomized trials. Furthermore, admittance to a NICU has been shown to reduce mortality and LOS and improve functional outcome as compared to a general ICU [2,17-19]. These benefits are most likely multifactorial and may be related to elevated attention in a neurocritical care setting to factors like reduced alertness that may result in secondary deterioration as well as neuroprotective measures such as normothermia, strict blood pressure management and management of cerebral edema formation.

In the present study we investigated the long-term outcome of treated patients receiving specialized neurocritical care and identified predisposing factors for a good functional outcome. As a key finding, overall outcome one year after treatment was fairly positive with 28.4% showing a good functional outcome of a mRS ≤ 2.

### Mortality

Mortality in critical care medicine is naturally high. Patients with hepatic encephalopathy (median age 58 years) showed a mortality rate one year after an ICU stay that amounted to 54% [20]. In critically ill surgical patients (mean age 65 years) survival one year after ICU discharge was reported to be 33% [21]. A systematic review of studies on general ICU patients showed a one year mortality between 26 and 63% (*n *= 5,725, mean age 55 years) [22]. Contrary, there are only limited data on outcome and mortality of NICU patients. The only available study that investigated a patient collective comparable to ours, reported a mortality rate of 47% after a mean follow-up time of 2.7 years [3]. In our cohort, overall mortality one year after NICU stay was as high as 39.8%. These rates were mainly driven by stroke patients and those with other diseases with high mortality rates such as neurodegenerative diseases, encephalopathy and inoperable cerebral neoplasms [23].

### Functional outcome and outcome-predicting diseases

Regarding the functional outcome of the surviving patients the parameter age is the major determinant of outcome, as described previously [3]. However, compared to many non-neurological diseases, age in neurological patients is not a fixed determinant for poor outcome but a rather relative parameter that has to be put into perspective to the underlying disease, for example, reversible inflammatory disease (GBS) *versus *irreversible brain tissue damage by stroke or ICH [3,6]. Several therapies applied in neurocritical care are linked to the age of patients. For instance, a 75-year-old patient with malignant middle cerebral artery infarction will not undergo hemicraniectomy because outcome likely will be poor with or without decompressive surgery, however, this is going to be investigated in the DESTINY 2 trial (ISRCTN21702227). In contrast, a patient with GBS of the same age will surely receive all possible critical care treatment. Given (i) an overall younger age of the analyzed patients of general and surgical ICU's as compared to our study (that is, approximately 60 years [20-22]* versus *67 years), and (ii) the age-associated co-morbidity of our patients, the functional outcome data presented here are respectable. In addition, functional outcome was significantly related to the severity of illness on Day 1 as reflected by the TISS-28, indicating that the severity of disease at admission predicts functional outcome one year after discharge. Therefore, the initial TISS-28 may be used to help decide whether invasive therapeutic procedures such as hemicraniectomy in malignant middle cerebral artery infarction ought to be carried out.

The admission diagnoses of GBS and myasthenia gravis were related to good functional outcome one year after discharge. Compared to patients with a benign disease course, patients with rapidly progressive neuromuscular weakness, dysautonomia and those requiring ventilation, thus requiring NICU admission, are known to have a less favorable outcome [24-26]. However, compared to irreversible central nervous system diseases, patients with GBS and MG who require neurocritical care tend to have a better functional outcome after one year. This is mainly based on the potentially reversible character of these diseases and the substantial progress in therapy within the last decades [27,28]. In contrast, patients with seizures did not reach statistical trends towards a good outcome. The finding that not all of the potentially reversible diseases showed a good outcome is most likely related to underlying severe co-morbidity [29,30], for example, symptomatic seizures after a stroke with a mortality of nearly 20% [31,32].

The diagnosis of ICH, often associated with intraventricular hemorrhage, was associated with both the highest in-hospital mortality and mortality after one year. This finding was in line with previous reports [3] and indicates that, while some patients with ICH improve clinically [33], the overall functional status of patients with this diagnosis deteriorates. ICH leads to substantial disability itself, causing reduced levels of consciousness, mechanical ventilation and extended ICU stay which again cause further complications, high early mortality and generally poor functional outcome [34,35].

### Length of ventilation

The LOV was independently predictive for a negative outcome. This feature has not yet been described. The length of hospital stay (LOS) showed a significant association with outcome in the univariate analysis but did not, as shown before [1,3,36], independently predict poor outcome and hence LOV, rather than LOS may serve as a surrogate marker for disease severity. This is most likely related to the fact that patients who required prolonged ventilation, per definition, had a longer LOS, whereas patients with prolonged LOS were not always mechanically ventilated and the LOS was occasionally affected by the availability of beds on other wards and rehabilitation centres. This aspect, that LOV rather than LOS reflects disease severity, might be focussed on in future studies as it appears likely that there are differences regarding the specific diseases.

### Limitations

The data presented have certain shortcomings, mainly the retrospective design of the study and the loss of some patients to follow-up analysis. Moreover, changes in staffing of our ICU might have altered treatment strategies and led to changes in outcomes. Outcome was assessed one year after neurocritical care, however, mailed questionnaires filled-out by patients or their relatives have an inherent predisposition for inaccuracy with respect to the validity of mRS estimation [37,38]. Further criticism might arise due to the rather rigorous cut-off rate we defined for outcome definition (that is, mRS ≤ 2 for good outcome). We aimed to identify only those patients who were functionally independent without requiring assistance for their daily activities. Other neurological studies which referred to the functional outcome as a primary endpoint have not necessarily used the same cut-off points [3,6]. Moreover, other important parameters possibly influencing outcome, such as GCS on admission or the requirement of intravenous catecholamines, were not sufficiently assessable in this retrospective analysis. Finally, the impact of rehabilitation was not assessed which may have influenced the results on functional outcome after neurocritical care.

## Conclusions

Although this appears counterintuitive, functional outcome and mortality of patients treated in specialized NICU units compare favourably with those in other intensive care fields. This highlights the impact of specialized neurocritical care procedures. The data presented here suggest that age, admission diagnosis, TISS-28 on Day 1, and length of ventilation are important independent predictors for outcome in neurocritical care patients. However, more disease-specific prognostic information on clinical course and functional outcome are needed to guide neurocritical care physicians in their identification process of patients who benefit from neurocritical care, and to possibly confine extended neurocritical treatment in certain situations, respectively.

## Key messages

• In neurocritical care, disease-specific prognostic information on clinical course and functional outcome are needed to guide neurocritical care physicians in their identification process of patients who benefit from neurocritical care.

• In this large, consecutive neurointensive care patient cohort, the diseases GBS and MG were independently related to a good long-term outcome, whereas older age and increased length of ventilation as well as the diagnoses ICH and cerebral neoplasm were predisposing factors for an unfavorable outcome.

## Abbreviations

DNT: do not treat; GBS: Guillain-Barré syndrome; GCS: Glasgow Coma Scale; ICH: intracranial hemorrhage; ICU: intensive care unit; LOS: length of hospital stay; LOV: duration time of ventilation; MG: myasthenia gravis; mRS: modified Rankin Scale; NICU: neurocritical care units; SAH: subarachnoid hemorrhage; TISS: Therapeutic Intervention Scoring System.

## Competing interests

The authors declare that they have no competing interests.

## Authors' contributions

ICK, SS and HBH designed the study and wrote the manuscript. PDS, MK and HBH performed substantial data extraction of institutional databases. JB, HL, SK and ICK reviewed the medical charts and obtained laboratory and radiological data. ICK obtained outcome data by mailed questionnaires and telephone interviews. PDS, MK and JB critically revised the manuscript. All authors approved the final version of the manuscript.
